# Diseases of Children

**Published:** 1894-03-17

**Authors:** 


### Medical Progress.

### DISEASES OF CHILDREN.

*Sporadic Cretinism.*—The treatment of myxœdema by means of the administration of thyroid glands or thyroid extract having proved so successful, attention was next directed to the employment of this new therapeutic agent in cases of sporadic cretinism[1]—the infantile form of myxœdema. A number of papers have recently been published on this subject, and the results obtained are in many cases very encouraging. Professor William Osler has made careful inquiry as to the prevalence of the disease in America, and finds that, contrary to the statements made by several writers, it is extremely rare in that country. Various forms of idiocy have been loosely described as of the cretinoid type, but the distinguishing features of the disease as regards the physiognomy, the shape of the head, the stunted growth, and the condition of the connective tissues have been absent. He agrees with Horsley in regarding the solid œdema of the skin and connective tissues as the most satisfactory diagnostic sign. Eleven cases are described, varying in age from three up to forty-two years, but in only one had the thyroid treatment been employed, and at the end of a month no special change was noticed. Dr. Byrom Bramwell has recorded a case of this disease in which the signs were extremely well marked. His patient was sixteen years old and 29½ inches in height. The affection had manifested itself at the age of nine months, after which mental and bodily growth had practically ceased. The accompanying photographs show a child of most repulsive aspect, and Dr. Bramwell says the appearance and cries of the patient were more like those of a lower animal than of a human being. Under the thyroid treatment she improved considerably; the œdematous infiltration disappeared entirely in a few weeks; and at the end of six months she had grown 6½ inches, she was more lively and intelligent, and looked two years older than before treatment. One very curious fact has been observed by Dr. Bramwell, namely, that in the part of Edinburgh in which this patient resided, cases of sporadic cretinism and myxœdema seem to abound. Within a radius of half a mile seven cases of those diseases have been discovered during the past year. We shall await with great interest the result of Dr. Bramwell's further investigations into this question of local causation. In the discussion which followed the reading of this case, it was pointed out that caution was required in carrying out the thyroid treatment, as rapid emaciation and great cardiac debility were apt to supervene, especially if the dose was excessive. Dr. J. Thomson has continued his notes of a case of sporadic cretinism which has been under treatment for twelve months. The patient was 18 years old, and measured 33½ inches in height. His growth had been at a standstill for 14 years, and since the thyroid treatment was begun he had gained 4⅝ inches. There is marked mental and bodily improvement, more especially the latter, and this is admirably shown in a series of photographs which accompany the paper. The author finds that there is no special advantage in frequent small doses of the thyroid gland or thyroid extract, and that the full effect is obtained by giving a dose twice a week. It is interesting to note by way of contrast to these results the utter hopelessness of such cases before the introduction of the thyroid treatment. Dr. J. A. Coutts saw a few years ago a patient, aged 24 years, who was one of the cases described in Dr. Hilton Fagge's original paper on sporadic cretinism. Sixteen years later he still presented the appearance of a child of two years, and, with the exception of a gain of two inches in height, his condition mentally and bodily was unchanged.

*Eye Diseases.*—An important communication has been made by Mr. Jonathan Hutchinson[2] on the subject of school ophthalmia. This disease, which has hitherto been prevalent in workhouse schools, has now appeared in high class schools as well, and in some cases as many as 40 to 50 per cent. of the children have been affected. The onset is insidious, unattended by much congestion of the ocular conjunctiva, and the disease in the initial stage is easily cured by local treatment (mercuric chloride or silver nitrate). The second stage is that of "follicular ophthalmia," and is not so amenable to treatment, while the third stage, that of "trachoma," frequently requires treatment extending over years. In all cases the patient should be isolated from the first, as the disease is considered to be epidemic in character, although evidence of direct contagion is usually obtained. In the treatment of acute gonorrhœal ophthalmia in children, Burchardt[3] has found the following method most useful. The head of the patient being thrown backwards, the conjunctival sac is freely irrigated with a 5 per cent. solution of chlorine water, followed by a 1-10 per cent. solution of nitrate of silver. At the same time both lids are moved up and down over the eye by the operator, so that the fluid penetrates to the remotest folds of the conjunctival sac.

*Tuberculosis.*—A case of general tuberculosis, in which the serous membranes were specially affected, is reported by Sergent.[4] The patient was a girl aged eleven years, who had suffered from loss of appetite and sickness, headache, emaciation, and debility. There was evidence of peritoneal and pleural disease, and she died after a series of convulsive seizures. At the necropsy two quarts of sero-purulent flocculent fluid were found in the abdominal cavity, and the peritoneum was lined in places with false membranes. A small quantity of sero-purulent material was present in the right pleural cavity, along with thickened pleural membranes containing granulations. The pericardium was distended by nearly two pints of hæmorrhagic fluid, and the membrane was lined with fibrinous exudation. Slight exudation was also present at the base of the brain, and a number of granulations were visible in the pia mater. Several cases of tuberculosis in which the infection was probably of intrauterine origin are described by Goldschmidt.[5] The children lived for sixteen, seven, and two months respectively. In all of them the tubercular lesions were so advanced, caseation and breaking down being present, that the writer concludes the disease must have been present at birth. Two similar cases are recorded by Thiercelin and Londe,[6] one child living for four days and the other for seven days. The mothers died of tuberculosis within a few days of their confinement. In one infant tubercle bacilli were found in the liver, spleen, and kidneys, although no naked eye changes were visible. Animals were inoculated with blood from the umbilical cord, and general tuberculosis was rapidly developed. In the treatment of tuberculosis, Dr. Clemente Ferreira[7] finds that creasote is well tolerated by infants, and that improvement usually follows its use. The maximum dose is seven grains per diem. Equally good results were obtained with guaiacol, of which as much as sixty grains may be taken daily. He has not found much benefit from aristol, iodoform, cantharidate of potash, or injections of chloride of zinc.

*Infant Feeding and Food Disorders.*—Budin[8] considers that the best test of an infant's nutrition is a steady increase in weight, as evidenced on systematic weighing. This increase, if the diet is suitable, should amount to a doubling of the weight in the first five months, and its trebling in the first year. He finds that the average daily gain is greatest in the case of breast-fed children, and when artificial feeding is necessary, he employs undiluted cow's milk which has been sterilised by raising it to a temperature of 212 deg. Fahrenheit in a steam bath. Similar conclusions as to the growth of children have been arrived at by Dr. Percy Boulton,[9] who holds that if a child does not increase in weight at the rate of 1 lb. a month during the first year of life, and 12 oz. a month during the second year, its nutrition is not satisfactory. Dr. Dwight-Chapin[10] points out that as milk is usually diluted when given to infants, it is necessary to retain as much of the fat in it as possible. With this object, at the New York Post Graduate Hospital, the milk is allowed to stand in deep, narrow vessels for three hours, after which the lower half is withdrawn. The part left contains 4·9 per cent. of fat (as compared with 3·1 per cent. in the former), and is then boiled in bottles plugged with cotton wool for fifteen minutes, so as to prevent fermentation. A note of warning has been sounded by Dr. Henry Koplik[11] on the subject of the habitual administration of alcoholic beverages to children, and their loose employment in various diseases. Many cases of cirrhosis of the liver have now been recorded due to this cause. In the case of illness, mothers are advised to give brandy or whisky, and if not expressly directed, will usually give it in excess, with the result that the child becomes restless and peevish, the appetite is lost, and a condition of great weakness supervenes. The dangers of feeding infants entirely on patent foods are pointed out by Dr. G. A. Sutherland[12] in an article on scurvy in children. This affection is traced to the excessive use of preserved or condensed foods which are now so much before the public, and does not appear in children who are fed on breast milk or sound cows' milk. Of seventy-one cases collected, fifty-seven occurred in children under two years of age. The leading signs, as contrasted with those present in the disease as it affects adults, are the presence of extensive hæmorrhage under the periosteum of the long bones, and the absence, in many cases, of sponginess and ulceration of the gums. Both of those conditions depend on the fact that the signs of rickets are usually well marked in these cases, and this affection produces an alteration in the local blood supply, that to the teeth being deficient, and that to the long bones being excessive. Meningeal and cerebral hæmorrhage are sometimes found. The treatment consists in the regulation of the diet, all patent and preserved foods being carefully avoided, and the administration of fresh fruits and vegetables, oranges, lemons, potatoes, &c. Medicines are not of much value, and mercury is often distinctly injurious.

*Abdominal Diseases.*—A discussion, introduced by Dr. Irving Snow, took place at the American Pediatric Society on the subject of gastric neurosis in children,[13] the symptoms of which in many respects resemble those of meningitis. Vomiting, gastric distress, constipation, and sometimes convulsions are present, but there are no physical signs of visceral disease. Excess of hydrochloric acid was present in the vomited material, but no benefit followed on the administration of alkalies. Rotch considered that the condition was due to reflex disturbance, and that the best treatment was to stop all food for twenty-four or thirty-six hours, and administer nerve sedatives by the rectum. Dr. Osler[14] has recorded two cases of dilatation of the colon in young children. The first patient, aged ten years, had suffered from constipation and great abdominal distension from infancy. Medical treatment affording no relief, laparotomy was performed, and although the colon was enormously dilated in its whole length, there was no obstruction found. An artificial anus was made at the sigmoid flexure. Relief followed the operation, and the patient gained flesh. The second case was that of a child aged seven months with distended abdomen. Visible peristalsis was occasionally present, and tarry motions were passed. The treatment ordered was the frequent passage of a catheter into the rectum, so as never to allow distension of the bowel to take place, but no improvement was noted after several months. The subject of acute diarrhœa in infants has been studied by Dr. R. E. Müller,[15] who regards the disease as due to ptomaine poisoning, the bacteria being present in the milk, and their toxic products being formed either outside of the body or in the intestinal tract. He recommends thorough irrigation of the bowel with cold or iced water. Dr. Fischl[16] also holds that infantile diarrhœa is a form of septicæmia, from a study he had made of twenty-one cases. Few micro-organisms were found in the intestine, but they were constantly present in the lungs, and also in the spleen, liver, and kidneys. He suggests that the organs primarily affected are the lungs. At a meeting of the Clinical Society, Mr. Malcolm[17] related a case of nephrectomy for malignant tumour in a female child twenty-three months old. He opened the abdomen in the right linea semilunaris, and after examining the condition of the left kidney, which was apparently healthy, he removed the right one along with the tumour through the posterior layer of peritoneum. A mass of glands around the right renal vessels was also cleared out. The wound healed well, and fourteen months later the child was described as being "in perfect health." On microscopic examination the neoplasm proved to be a "malignant adenoma, and the glands showed no secondary deposit.

*Other Diseases.*—Tissier[18] has pointed out that infants may suffer from a purulent rhinitis, strictly analogous to purulent ophthalmia neonatorum, and due probably to the same cause—namely, infection from the maternal passages. He, therefore, advises careful cleansing of the nostrils with some antiseptic solution after birth, in all cases where the mother is known to have a vaginal discharge, and if purulent rhinitis developes, applications of a solution of silver nitrate (one in twenty). During an epidemic of measles, Dr. C. E. Shelly[19] tried the treatment by eucalyptus inunction in five cases. The patients were kept together in one ward, and were in no way selected. Oleusaban was rubbed over the body night and morning for three days, and subsequently once a day for the first week. Amongst the results noted were drowsiness and dulling of sensibility, abnormally furred tongue, relatively prolonged pyrexia, and delayed eruption. Dr. Shelly does not find his results encouraging, but believes that the treatment employed in the other cases—namely, ten-grain doses of benzoate of sodium, proved distinctly beneficial. Dr. Koplik,[20] in a paper on malarial fever in infants and children, remarks that all the cases he had met with in New York had either lived out of the city or had been abroad, and further that in some of his cases the signs of malaria did not appear until the patients had left the country and been resident in town. The tertian type is the most common, being present in thirteen out of fifteen cases. The plasmodium malariæ is usually found in the blood. In the treatment of this affection, he recommends the bisulphate or hydrochlorate of quinine, which children tolerate well, and later on the administration of arsenic until the spleen is normal in size.

*The After Effects of Chloroform.*—Dr. L. G. Guthrie[21] has drawn attention to a hitherto undescribed condition in children after prolonged chloroform anæsthesia. The patient may recover in the ordinary manner, but within a few hours alarming symptoms supervene, namely, wild screaming delirium, restlessness, sleeplessness, and incessant vomiting of peculiar character. Ten cases of this nature are described, nine of which proved fatal. In a careful and exhaustive discussion of the possible causes of this condition, Dr. Guthrie mentions: (1) Shock, (2) carbolic acid poisoning, (3) iodoform or mercurial poisoning, (4) pre-existing pathological conditions, and (5) the remote effects of chloroform, and by a method of exclusion, is compelled to ascribe the symptoms to the last-mentioned. As regards the pathological changes present, he found that in most of the cases the liver was infiltrated with fat, and as chloroform is known to increase this condition, he infers that death was due to poisoning from toxic alkaloids set free in the system, and not eliminated by the kidneys. These alkaloids would, under normal conditions, be prevented from entering the general circulation by the action of the liver, which passes them into the bile, but if that organ is functionally impaired, as by the condition of fatty infiltration, symptoms of poisoning at once take place. No treatment seemed to have any effect in controlling the symptoms, and most of the cases ended fatally within twenty-four hours of the operation. Mr. Watson Cheyne,[22] who operated in several of the cases referred to, is of opinion that death was due, in the case of his own patients, to carbolic acid poisoning in some, and possibly fat embolism in others. He points out that most of the deaths occurred at a time when carbolic acid was used very freely both before and during an operation, and since he has employed it sparingly he has met with no more cases of a like nature. The question is one of great importance, and for its settlement we must wait for further information.

[1] Osler, Am. Jour. Med. Sci., Nov. 1893, p. 503; Byrom Bramwell. Brit. Med. Jour., Jan. 6, 1894, p. 6; Discussion on above, Ed. Med. Journ., Feb. 1894, p. 742; Thomson, Ibid, p. 720; Coutts, Brit. Med. Jour., Jan. 27, 1894, p. 186. [2] Brit. Med. Journ., Feb. 3, p. 243, and Feb. 10, p. 329. [3] Centralbl. f. prakt. Augenheilk., Nov., 1893, and Epitome B. M. J., Jan. 6. [4] Am. Jour. Med. Sci., Nov., 1893, p. 601, and Bull. de la Soc. Anat. de Paris, t. vii., ser. v., f. 15, p. 361. [5] Münch. Med. Woch., Dec. 26, 1893, and Ep. Brit. Med. Jour., Jan. 27, 1894. [6] Fortsch. d. Medicin, Jan. 15, 1894, p. 61. [7] Bullet. gen. de Therap., 1893, 28 livr. p. 68, and Am. Jour., Med. Sci., Nov., 1893, p. 593. [8] Rev. Gen. des Sciences, Nos. 21, 23, 1893, and Ep. B. M. J., Jan. 13, 1894. [9] Brit. Med. Jour., Jan. 27, 1894. [10] New York Med. Jour., Sep. 16, 1893, and Med. Chron., Jan., 1894. [11] Med. News, II., 1893, p. 481, and Ep. B. M. J., Feb. 3, 1894. [12] Practitioner, Feb., 1894. [13] Archiv. of Pediat., Dec., 1893, and Ep. B. M. J., Jan. 27, 1894. [14] Archiv. of Pediat., Feb., 1893, and Med. Chron., Jan., 1894, p. 246. [15] Therapeutic Gazette, Nos. 7 and 8, 1893, and Am. Jour. Med. Sci., Nov., 1893, p. 591. [16] Deut. Med. Woch., Nov. 9, 1893, and Ep. B. M. J., Dec. 9, 1893. [17] B. M. J., Feb. 3, 1893, p. 242. [18] Rev. des Mal. de l'Enf., Jan., 1894, and Ep. B. M. J., Jan. 27, 1894. [19] The Practitioner, Nov., 1893, p. 321. [20] New York Med. Jour., Sep. 6, 1893, and Med. Chron., Jan., 1894. [21] The Lancet, Jan. 27 and Feb. 3. [22] The Lancet, Feb. 10, p. 370.

